# Unusually High Levels of CA19-9 Associated with Mature Cystic Teratoma of the Ovary

**DOI:** 10.1155/2014/187910

**Published:** 2014-09-08

**Authors:** Monika Madaan, Manju Puri, Ritu Sharma, Harvinder Kaur, Shubha Sagar Trivedi

**Affiliations:** ^1^LHMC and SSKH, New Delhi 110001, India; ^2^ESIC Hospital, Manesar, Haryana 122001, India

## Abstract

*Introduction.* Mature cystic teratoma is the benign tumor of the ovary. CA19-9 levels, although a marker of pancreatic malignancy, have been found to be raised in large ovarian mature cystic teratomas. *Case Report.* We report a case of a young female who had unusually high levels of CA19-9 in the blood associated with large ovarian mature cystic teratoma. The levels returned to normal 12 weeks after cystectomy was performed. *Conclusion*. This case highlights the fact that although raised tumor marker may be associated with a benign pathology thorough evaluation to rule out malignancy still must be done.

## 1. Introduction

Mature cystic teratoma or dermoid tumor is the most common tumor of young females and constitutes up to a third of all benign ovarian tumors. It is composed of well-differentiated tissues derived from all three germ layers. It is commonly diagnosed based upon clinical findings, ultrasonography, or CT scan. CA19-9 levels are raised in patients with malignancy of pancreas, biliary tract, colon, esophagus, and liver carcinoma. There are reports in the literature documenting raised blood levels of CA19-9 in women with mature cystic teratomas but there is no report associating extremely high levels with a benign ovarian pathology. We describe a case of mature cystic teratoma associated with very high levels of CA19-9 in the blood.

## 2. Case Presentation

A 27-year-old woman, married for 9 months, P0A1, was referred to us in the gynaecology outpatient clinic with the diagnosis of ovarian cyst on USG and CA19-9 level of 1826 U/mL. She complained of heaviness of the lower abdomen and gradually developing distension for the past 4 months. Her menstrual cycle was normal and past history was not significant. On examination, her vitals were stable and abdominal examination revealed a large intra-abdominal mass reaching up to the umbilicus. Vaginal examination revealed normal sized retroverted uterus deviated to the right. The mass felt per abdomen could be tipped through anterior and left fornix. The movements of the mass were not transmitted to the cervix. In investigations, her hematological and biochemical profile was normal. Transabdominal USG showed a complex cystic lesion measuring 15 × 13 × 9.5 cm in the left ovary with internal echoes and septae, giving the possibility of mature cystic teratoma. CT scan also reported the cyst as left ovarian dermoid. The other abdominal and pelvic organs were unremarkable. In view of abnormally high values of CA19-9 in association with ovarian cyst other tumor markers like CA125, AFP, LDH, and beta hCG were measured and were found to be normal.

Patient was taken up for exploratory laparotomy. A large left sided ovarian mature cystic teratoma measuring around 15 × 12 cm was present. Uterus, bilateral tubes, and right ovary were normal. Left sided ovarian cystectomy was done. The cyst was removed intact; there was no extracapsular extension, no spread to the other ovary, and no ascites. A thorough exploration of the whole abdomen was carried out and no abnormality was detected. Frozen section of the cyst was reported as mature cystic teratoma and the tumor was staged as stage 1. CA19-9 levels decreased to 975 U/mL 5 days after surgery and finally returned to normal 2 months following surgery.

## 3. Discussion

CA19-9 is a sialylated Lewis blood group antigen which is primarily used for diagnosis, follow-up, and prognosis of pancreatic carcinoma. Its normal value is <35 U/mL. It is considered as the most sensitive and specific marker of pancreatic carcinoma. Its level is elevated in malignancies of biliary tract, colon, esophagus, and liver. Certain benign conditions like pancreatitis, biliary disease, and cirrhosis are also associated with elevated levels >1000 U/mL [1]. However 5–7% of population is Lewis antigen negative and in them CA19-9 levels are undetectable in the blood irrespective of the pathology [1].

Increased levels of CA19-9 have been observed in some cases of mature cystic teratoma. Atabekoglu et al. described a case report with CA19-9 value of 1430 U/mL with dermoid cyst [2]. Their study also showed that it is secreted from the apical cytoplasm of the epithelial lining of the cyst wall as is shown by immunohistochemical staining resulting in detectable levels found in systemic circulation. Abnormally high levels have been found in very large mature cystic teratomas probably associated with inflammation of the cyst wall or a weakened cyst wall. This also explains very high levels found in our case as the size of the cyst was quite large and also the reason for the long time taken for the levels to return to normal. Coskun et al. described mean levels of 109.1 U/mL in their case series of 43 patients with an average diameter of 7.7 cm [3]. Kikkawa et al. found the mean level of 217.6 U/mL in their series of 71 cases [4].

Brain et al. described markedly high levels of 2880 U/mL in a case with ruptured ovarian cyst (mucinous cystadenoma) associated with ascites [5]. Studies have shown that CA19-9 could be increased in up to 50% of cases of immature cystic teratoma [6]. It can be used as a marker for postoperative follow-up in benign disease and as a marker for recurrence of mature cystic teratoma.

## 4. Conclusion

To conclude, extremely high levels of CA19-9 may be associated with benign ovarian pathology, but thorough preoperative workup for any evidence of malignancy is a must.
